# Some Lithuanian ethnobotanical taxa: a linguistic view on Thorn Apple and related plants

**DOI:** 10.1186/1746-4269-2-13

**Published:** 2006-03-02

**Authors:** Daiva Šeškauskaitė, Bernd Gliwa

**Affiliations:** 1 Kaunas College of Forestry and Environmental Engineering, Girionys, Lithuania; 2 Sargeliai, Lithuania

## Abstract

**Background:**

The perception and use of plants correspond with common plant names. The study of plant names may give insight into historical and recent use of plants.

**Methods:**

Plant names in dictionaries and folklore have been evaluated. A etymological analysis of the names is provided. Onomasiological and semasiological aspects have been considered. Therefore, species named with names related to each other have been selected.

**Results:**

Plant names containing the stem *dag*- or *deg*- may belong to either of two categories: incenses or thorny plants. Plants named in *durn*- have been in use as psychopharmaca. The name *rymo *points not to Rome but to the use of plants as anodyne or psychopharmaca.

## Background

The study of plant names is a quite old branche in ethnobotany. It is connected with comparative historical linguistics and Indo-European studies [1] or philological studies of singular languages, e.g. the dictionary of German plant names [2]. Names correspond with the perception and categorisation of plants. This is true for the binary scientific names [3] as well as for common plant names.

Linguistic studies have shown that plant names of common origin may refer to different plant species. E.g. Latin *fāgus *'*Fagus *sp.' vs. Old Greek *phēgós *'*Quercus *sp.'. Various explanations have been given, the most frequent is probably the missing of a certain plant in the area where speakers of a language came to settle. Another view points to an open question: we do not know what the reconstructed Indo-European *bhago- really meant. It could name *Fagus *sp. as well as any tree with leaves or hard timber [4,5].

## Methods

Lithuanian plant names have been selected from written sources and common use. A philological analysis of the names has been provided, their botanical meaning has been discussed. Both, semasiological and onomasiological aspects have been considered. Therefore, species with identical or similar names have been selected for the present study. The etymological analysis is related with plant names from neighbouring Slavic and Germanic languages. It is supposed that names correspond with the use of these plants in traditional medicine, rites and handicrafts. Ethnographic data about plants have been an important source for the study. Due to requirements of linguistic papers we italicise not only botanical taxa but also linguistic forms. We hope that this will not result in confusion. Diacritics have been omitted.

## Results and discussion

### Durna

*Durnaropė *ist the most popular Lithuanian name for *Datura stramonium*. It is also used for recently introduced ornamental plants of the genus *Brugmannsia*. Other names for *D. stramonium *are *durnadagilis*, *durnadagis*, *durnarėjus*, *durnažolė*, *durniukas *etc. [6,7]. Linguists compare these terms usually with Russian *durman *'*D. stramonium*' and *durnyj *'stupid' and claim that the Lithuanian plantnames and Lithuanian *durnas *'stupid' have been borrowed from Slavic languages [8]. The origin of *D. stramonium *remains unclear until now. The species is indigenous either to Central America or to Asia or cosmopolitan. No sure evidence (palynolonogy, macro fossils) for neither views has been provided so far. It seems rather impossible to falsify one of these opinions. Therefore, linguistic discussion has no firm basis when it regards only this species. Additionally, there are other plants named in a similar way: *durnažolė*, *durnius *'*Hyoscyamus niger *L.', *durnažolė *'*Atropa bella-donna *L.', *durnarepka *'*Scopolia carniolica *Jacq.' and *durnažolė *'*Lolium temulentum *L.' [6,9]. All species are psychoactive. No doubt, the names refer explicitly to this effort. *Hyoscyamus niger *and *Lolium temulentum *are indigenous plants or at least archeophyts.

The use of psychoactive plants is a human universality and-generally speaking-not a matter of loaning. If plant names in *durn*- were loans then there should be synonymous names with Lithuanian *paikas*, *kvailas*, *žioplas*, *trenktas *etc. 'stupid, crazy'. In fact, only a single *žioplys *'idiot' appears for *D. stramonium*. Not only therefore, the conclusion is that *durnas *'stupid' is not a loan but Baltic heritage, however, cognate with the Slavic words for narcotic plants, e.g. Russian *durman*, Belorussian *durzilje *'*D. stramonium*', Russian *durnišnik *'*Xanthium *L.', Ukrainian *dur *'*Hyoscyamus niger*' [7].

A considerable number of Lithuanian phrases concern henbane, thorn apple etc. and their well known effort: "The drunkard gambols like having consumed henbane", "he talks nonsens as if he would have eaten thorn apple", "he turns around as if he would have consumed thorn apple" etc. [10]. The effort is temporary and primarily not a matter of character or intellect. Therefore, it is suggested that the original meaning of *durnas *etc. was not 'stupid' but 'drunken, high'. The use of psychoactive plants belongs to the responsibility of religion in quite a lot of cultures [11]. One can suppose that pre-Christian local religions used them as well. A hint is the name *dievažolynis *'God's herb' for *D. stramonium *[10], where *dievas *'God' is probably the Pagan god of heaven, predecessor of the Christian God, for the Catholic rite do not favour the use of narcotic plants.

### Rymo

Several Lithuanian plant names contain the word *Rymo*: *Rymo ridikas*, *Rymo ropikės *'*Atropa bella-donna *L.', *Rymo ropė *'*Datura stramonium *L.' and '*Scopolia carniolica *Jacq.', *Rymo ramunė *'*Matricaria recutita *L.' [6,10]. The spelling shows that *Rymo *has been understood as proper name: *Rymo *'of Rome'-Old Lithuanian *Rymas*, Mod. Lith. *Roma *'Rome'. Regarding origin, range and use of these plants one misses a clear motivation for this label [12]. Generally, *ridikas *refers to any tap-root, *ropė *names turnip, tubers, knobs-roots as well as fruits.

The origin of *D. stramonium *is not clear as mentioned above, but Rome is no serious option. For *Scopolia carniolica *the species designation *carniolica *refers to its origin respectively area of first description in Carniola. Russian belladonna occurs wild in the Carpathians, Caucasus, Alps [11]. Others note its spreading from the Apennine peninsula to East Europe [13] and a cultivation in Lithuania, Latvia and East Prussia and its use as narcotica, aphrodisiaca and for healing nervous fits [14]. However, *S. carniolica *has not even been mentioned by Dagys [6]. *Atropa bella-donna *grows wild in most parts of Europe, South East Asia and North Africa [13]. *Matricaria recutita *is an indigenous plant to Lithuania, now rather rare [15].

The species of the family *Solanaceae *under discussion have in common that they are introduced to Lithuania. That would it make possible to receive a geographic attribute as, to give an example, *graikiškas riešutas *'Greek nut' *Juglans regia *L. did. The latter agrees with the way the plant was cultivated and spread. The Persian Walnut is very often attributed with geographical terms [2]. On the contrary, the plants attributed in *Rymo *have no names in neighbouring languages referring to their origin, except Russian belladonna with a different localization, however. A recent study has shown that common Lithuanian plant names-not book names-refer very seldom to the geographic origin of the plant [16].

We suggest that *rymo *(genitive of *rymas*) has nothing in common with Rome. It is by accident phonetically equal to the Old Lithuanian name of Rome: *Rymas*. Therefore the option to reinterpret the meaning of the word-a folk etymology-has been given. But what is the origin of *rymo*? Have a look on data of Lithuanian lexicology and folklore.

A once popular Lithuanian game is named "*ropę rauti*" 'to root out the turnip' [17]. One actor explains that his wife has a violent urge for *Rymo ridikas*, therefore it should be rooted out right now [18]. Another version requires *trimo lapo *'stanching leaf', because the actor has been bitten by a dog.

The urge for *Rymo ridikas *appears in phrases such as *Užsigeidė kaip bobutė Rymo ridiko *'She demands rymo turnip like an old woman' [17]. Noteworthy, in all versions of the phrases female persons require it. This circumstance makes it less convincing to argue with narcotica in this special case. The game is typical for wedding ceremonies, the man is looking for medicine for a female person. It could be supposed that the game was the modelling of birth and asking for the midwife. Therefore-asking the midwife-special phrases have been used, straightforward terms like child giving or birth were to avoided, usual encodings were e.g. *pečius sugriuvo *'the stove tumbles down', *gandras atnešė broliuką *'the stork delivered a brother' [19].

*Scopolia carniolica *and *Hyoscyamus niger *are known for traditional use aborning to soothe the pain in labour [9,20]. *D. stramonium *and *A. bella-donna *are also anodynes. Data on real or possible use of the latter both species while child giving we did not observe. All plants in *rymo *are officinal ones. Lithuanian *raminti *'to calm, quieten; soothe; console; ease pain' is a favourite word to express soothing. Related words are e.g. Lithuanian *ryma *'tranquility, calm', *rymoti *'to think, meditate', *ramus *'quiet' [8,10]. There is no hindrance to integrate *Rymo *and all of the plant names into this word family.

*Rymo ramunė *'*Matricaria recutita*' is said to ease pain in the stomach: *ramuliai vidų ramina *[10]. Therefore, we suppose that not only *rymo *but also *ramunė *belongs to the family of *rimti *'to became quiet, calm' as well. Linguists claim that Lithuanian *ramunė *etc. and others like Russian *romaška *'*Matricaria *spp. etc.' goes back to Latin *anthemis romana*, *chamaemelum romanum *[8,21].

However, the origin of this Latin name shows that it has nothing in common with Rome. Marzell [2] notes that *romanum *only means 'not native' here; but this is not the point. He remarks further that the claim mentioned by Mattioli in 1600 "the species grows around Timur near Rome" triggered off the use of *romanum*. In fact, it did not grow there at this time, it was unknown to authors of antiquity [2]. So, why did Mattioli claim this? The answer lies in this case in common German names, e.g. *Romei, Remey *'*Matricaria *spp., *Chamaemelum *spp.' Relating the name which sounds like Rome to Rome is a usual linguistic process named folk etymology, the same as we report for Lithuanian *Rymo*.

The situation with the Russian material is very similar. Merkulova [21] notes Russian *cvetu romanova *'Roman flower' and *romanova trava *'Roman herb' found in written sources from 1534. She claims that Middle Age Latin called the plants *anthemis romanum, chamaemelum roman*, unfortunately without giving evidence for this claim. This would classify the names as pure book names. The Russian and Polish spelling of Rome is with *i *or *y*. Versions with *o *are quite new-from the 20^th ^century. So we have the strange situation that Russian *rimskaja romaška *'*Chamaemelum nobile*' would point to Rome in both parts. The older one (*romaška*) would show the modern spelling of Rome. The new one, which is indeed a translation from a binary (Latin) plant name, shows the traditional spelling.

Therefore we conclude that Russian *romaška *as well as Lithuanian *rymo*, German *Romei *refers to the healing properties of *Matricaria recutita*. All these plant names should be cognate to each other. Similar plant names in *rymo *for *Atropa bella-donna, Scopolia carniolica *and *Datura stramonium*, on the one hand, and *Matricaria recutita*, on the other hand, result from the use of *rimti *for both, haemostatic effort on wounds (cf. German *die Blutung einer Wunde stillen *'to staunch a wound' where *still *means 'calm, quiet') and the effort on the psyche.

### Dagys

Lithuanian plant names containing the stem *dag*- or *deg*- may belong to one of three different concepts. However, only one of them is taken into consideration usually i.e. the concept of thornes, spines and prickles: Lithuanian *dagys *and *dagilis *'*Carduus *spp., *Cirsium *spp., *Onopordum acanthium *L., *Silybum marianum *Gaerth.', *dagišius *'*Xanthium spinosum *L.', *dyguldagis *'*Datura stramonium *L.' [6,8,10]. One more example, where *dag*- refers to the concept of thorns or the like are *dagutis*, *diegiažolė*, *dygulis *etc. '*Geum urbanum *L.'. The plant has no thorns but the fruit is prickly like burdock's one. Lithuanian *velniadagis *'diablo's prick' names *Arctium *sp.

The second concept refers to Lith. *degti *'to burn' [22]. *Deguèiukas *'*Lychnis viscaria *(L.) K. Jess' is related as well. This name, however, should be seen in relation to the use of stalks made from *Lychnis *spp. for candle wicks [2]. *Lychnis *had been derived from the Greek name for light as well [3].

A third concept has been introduced recently [7,23]. It is similar to the preceding one. It refers to a use as incense especially in healing.

Additionally, Lithuanian *dagilis *appears in refrains of of Lithuanian folk songs "*Lio lelijėla, lio dagilio; Lelijėla, dagilio*", "*Dobile, dogile, Dobile, totata*" [23]. Here, the term is connected with other plant names: *lelija *'lily' and *dobilas *'clover'. It is very difficult to find out which species is meant by *dagilis*. As we can exclude any reference to thorns, it seems possible to suggest a use as incense, e.g. for healing purposes.

In order to heal the folk illness timorousness (Lithuanian *išgąstis*) the patient is smoked with seeds of *Datura stramonium *L., flowers of *Paeonia *spp. and *dagis *[9], with dry grinded *dagiliai *[24]. The identity of the latter-*dagis *and *dagiliai*-remains unclear.

Different interpretations seem applicably for *durnadagis, dyguldagis *'*Datura stramonium*'. It has been named thorn apple with respect to its thorny fruit. The use as incense has been attested: throwing *D. stramonium *on the hot sauna oven has been an erotically charged joke somewhere in rural Lithuania [11,25]. Common German plant names *Rauchöffelkraut, Asthmakraut *'*D. stramonium*' [2] refers also to healing properties.

Common names of *Potentilla erecta *(L.) Raeusch. are beyond question. *Degimai *'burning', *degimo žolė *'burning herb', *degsnys *'burnt place' refer to words for burning; *dagiai *and *dagiukas *are suspected to do so as well. The plant was used as incense as can be seen from its name *priemetžolė *'herb for nervous fits'. The illness *priemėtis *is widely healed smoking herbs.

## Conclusion

Plants may-according to use and habit-belong to different ethnotaxa. This has been shown for three Lithuanian categories, each of them containing *Datura stramonium*. The affinity of a certain plant to a category is sensitive to the language used in the name-giving process-only a language where one word is appropriate for stanching wounds, easing pain and calming the psyche may group *Atropa bella-donna, Scopolia carniolica, Datura stramonium *and *Matricaria recutita *together.

## Competing interests

The author(s) declare that they have no competing interests.
